# Fcγ Receptors in Solid Organ Transplantation

**DOI:** 10.1007/s40472-016-0116-7

**Published:** 2016-10-03

**Authors:** Tomas Castro-Dopico, Menna R. Clatworthy

**Affiliations:** Molecular Immunity Unit, Department of Medicine, MRC Laboratory of Molecular Biology, University of Cambridge, Francis Crick Avenue, Cambridge Biomedical Campus, Cambridge, CB2 0QH UK

**Keywords:** Antibodies, IgG, Fcγ receptors, Antibody-mediated rejection, Single nucleotide polymorphisms, Infection

## Abstract

In the current era, one of the major factors limiting graft survival is chronic antibody-mediated rejection (ABMR), whilst patient survival is impacted by the effects of immunosuppression on susceptibility to infection, malignancy and atherosclerosis. IgG antibodies play a role in all of these processes, and many of their cellular effects are mediated by Fc gamma receptors (FcγRs). These surface receptors are expressed by most immune cells, including B cells, natural killer cells, dendritic cells and macrophages. Genetic variation in *FCGR* genes is likely to affect susceptibility to ABMR and to modulate the physiological functions of IgG. In this review, we discuss the potential role played by FcγRs in determining outcomes in solid organ transplantation, and how genetic polymorphisms in these receptors may contribute to variations in transplant outcome.

## Introduction

Immunoglobulin G (IgG) antibodies are the most abundant immunoglobulin isotype in human serum and extracellular tissue fluid. They play an important role in defence against infection via pathogen neutralisation and opsonisation and complement activation, and can directly stimulate a wide variety of immune cells by cross-linking cell surface Fc gamma receptors (FcγRs) [1, 2]. However, autoantibodies are pathogenic in a number of autoimmune diseases [3, 4] and in solid organ transplantation alloantibodies are associated with antibody-mediated rejection (ABMR) [5, 6]. Indeed, in the current era, the presence of donor-specific anti-HLA antibodies (DSA) represents a major hurdle in transplantation. Sensitised transplant recipients with pre-formed DSA now make up a third of wait-listed kidney transplant recipients, and have a significantly increased risk of acute and chronic ABMR, resulting in reduced allograft survival [7, 8]. In non-sensitised subjects, the development of de novo DSA is also associated with worse outcome, particularly if they occur many years after the transplant [9, 10].

**Fig. 1:**
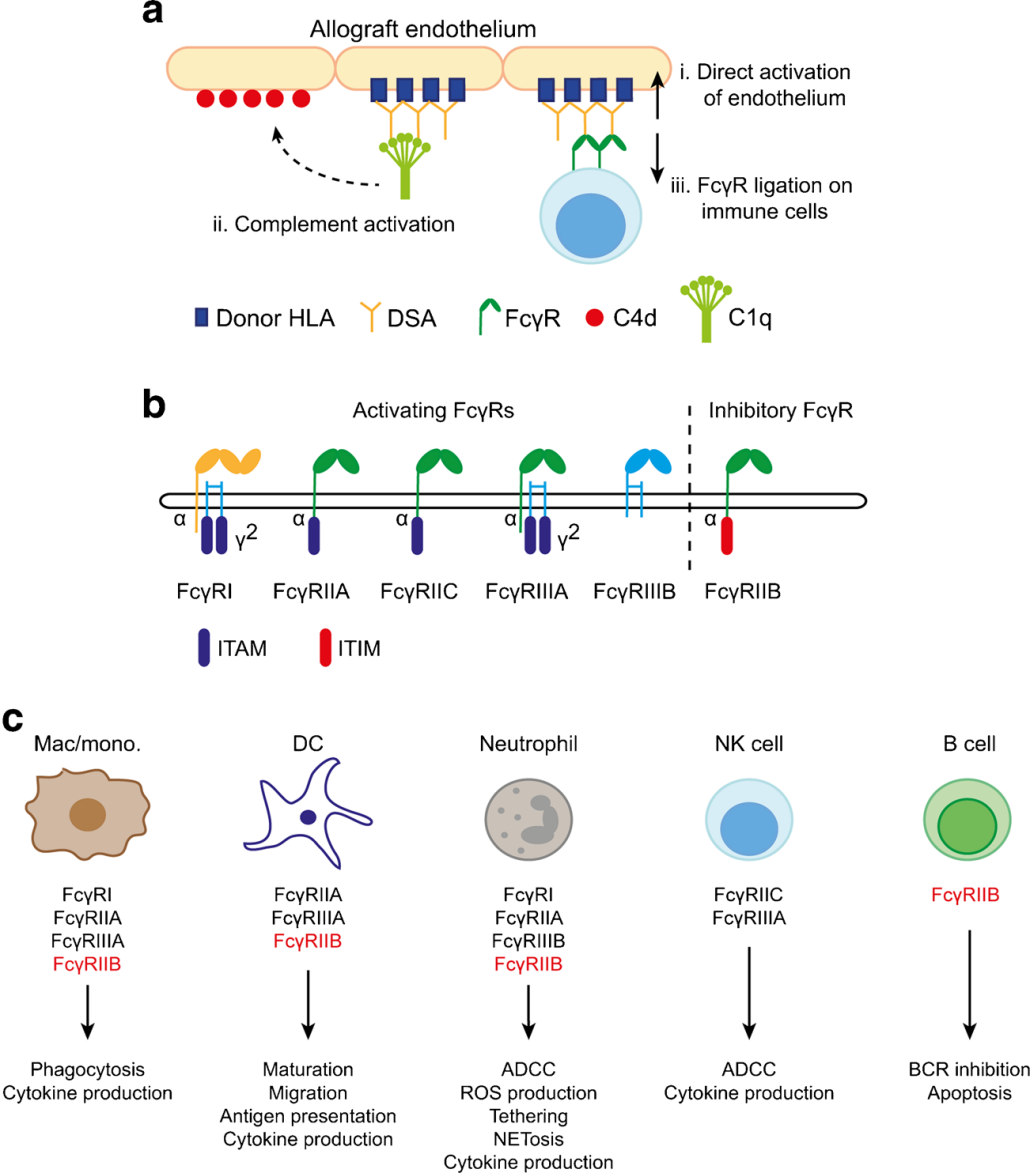
Human Fcγ receptors. **a** FcγRs in antibody-mediated rejection. DSA deposition within allografts can stimulate numerous pro-inflammatory mechanisms, including the direct activation of graft endothelium (*i*), complement activation via the classical pathway (*ii*), and the activation of FcγR-expressing immune cells. **b** Human FcγRs family members differ in IgG affinity, cellular distribution and signalling mechanisms. There are five activating FcγRs that signal via immunoreceptor tyrosine-based activation motifs (ITAM), four with low IgG affinity (FcγRIIA, FcγRIIC, FcγRIIIA and FcγRIIIB) and one with high affinity (FcγRI), capable of binding monomeric IgG. There is a single inhibitory receptor, FcγRIIB, with an intracellular immunoreceptor tyrosine-based inhibitory motif (ITIM). **c** Cellular distribution and function of FcγRs—FcγRs are expressed across numerous immune cells implicated in ABMR, and promote cell type-specific immunological mechanisms that could contribute to allograft rejection, including endothelial adhesion, ADCC, pro-inflammatory cytokine production and ROS production

With accumulating evidence of the deleterious effects of IgG DSA on long-term allograft survival, there has been increased interest in understanding the mechanisms that drive tissue damage in the context of ABMR. The observation of CD4d deposition in the peritubular capillaries of biopsies with histological changes of ABMR and DSA led to the assumption that complement activation plays a key role in antibody-associated allograft damage. However, the absence of C4d staining in more than half of biopsies with late ABMR highlights the importance of complement-independent mechanisms in mediating the deleterious effects of DSAs [11, 12]. Furthermore, some IgG isotypes (IgG4) cannot fix complement, whilst IgG2 has a limited complement-activating capacity compared with IgG1 and IgG3 [13]. Reed and colleagues have produced an elegant body of work demonstrating that HLA antibodies can have direct effects on allograft endothelial cells via variable region binding [14], but the engagement of FcγRs on immune cells and on endothelium is also likely to be of critical importance in generating alloantibody associated inflammation (Fig. 1a). FcγRs bind to the Fc portion of IgG and mediate the activation of both innate and adaptive immune cells. Variation in the genes encoding these receptors can alter IgG binding to FcγRs and receptor activity, and may therefore influence the magnitude of inflammation induced by alloantibodies as well as the risk of developing alloantibodies.

Although chronic ABMR represents a major challenge to graft survival, one of the commonest causes of graft loss in the long-term is death of the transplant recipient with a functioning graft. This most frequently occurs in the context of infection, malignancy or cardiovascular disease, all of which may be influenced by humoral immunity. In the case of infection and malignancy, IgG antibodies may have beneficial effects [15, 16], whilst in atherosclerosis their function is less clear [17–19], with FcγRs again playing a key role.

In this review, we discuss the impact of FcγRs on immune cell activation and consider their potential impact in transplant rejection and recipient survival. Given the clinical and therapeutic similarities between ABMR and IgG-mediated autoimmune diseases, such as systemic lupus erythematosus (SLE), many of the data identifying the cellular mechanisms underpinning the pathogenic effects of antibody may inform discussions. We will therefore also consider this evidence, where relevant to antibody-mediated pathology in transplantation.

## Fcγ Receptors—Overview and Cell Distribution

FcγRs are cell surface molecules that bind to the Fc portion of IgG antibodies to initiate intracellular signalling pathways, leading to immune cell maturation and activation. In humans, there are several activating receptors (FcγRIIA, FcγRIIC, FcγRIIIA and FcγRIIIB) and a single inhibitory receptor, FcγRIIB, which plays a critical role in suppressing IgG-mediated inflammation [1, 20] (Fig**.** 1 **b**). FcγRs are widely expressed on immune cells, including neutrophils, monocytes, macrophages, dendritic cells (DCs), mast cells, natural killer (NK) cells and B cells, but the type of FcγRs expressed differs between cell types (Fig. 1c). Notably, T cells do not express FcγRs. In addition to binding IgG, FcγRIIA can also bind to acute phase response proteins, C-reactive protein (CRP) [21•] and serum amyloid P (SAP) [22].

Most FcγRs are low-to-medium affinity for IgG, requiring cross-linking of several receptors into signalling synapses on the cell surface in order to initiate productive signalling. This is achieved through the formation of high avidity immune complexes (IC) between antigen and antigen-specific IgG or by IgG-opsonised cells. The absence of signalling upon ligation of monomeric IgG prevents inappropriate immune cell activation, which is critical, given the abundance of circulating monomeric IgG. The inhibitory receptor, FcγRIIB, acts as an additional regulatory mechanism to suppress IgG-mediated inflammation, although its expression is heterogeneous across cells of the immune system and subject to regulation by various stimuli, particularly by the cytokine milieu [23, 24]. For example, Th2 cytokines such as IL-4 and IL-33 increase monocyte expression of FcγRIIB [23, 25••], whilst interferon-γ (IFNγ) leads to a reduction in FcγRIIB expression on monocytes and DCs [26]. The ratio of activating to inhibitory FcγRs on any given cell is known as the activating/inhibitory (A/I) ratio, and its context-specific modulation allows for appropriate immune responses to be raised [1, 27]. Genetic polymorphisms in human *FCGR* genes that alter receptor expression or function are frequently associated with differential susceptibility to both infection and autoimmunity [15, 20, 27]. Genetic variation in FcγRs is not the only factor that influences the outcome for a cell encountering IgG immune complexes; differences in IgG glycosylation can alter affinity for activating versus inhibitory FcγRs [28–31]; for example, de-fucosylation increases the binding affinity of IgG for activating FcγRIIIA (but not FcγRIIB) 10–50 fold [32]. Data indicate abnormalities in the IgG glycome in some patients with SLE, with a reduction in galactosylation and sialylation of IgG that might potentially favour binding to activating FcγR [33]. A reduction in galactosylation has also been observed in patients with rheumatoid arthritis [34], but there is currently no information on whether differences in the glyosylation state of DSA might impact their pathogenicity.

### FcγR Signalling

Activating FcγR cross-linking leads to tyrosine phosphorylation of the immunoreceptor tyrosine-based activating motif (ITAM) within the associated common Fcγ chain by the Src-kinases Lyn and subsequent recruitment of SH2-containing kinases [35]. This ultimately leads to the activation of phosphatidylinositol3-kinase (PI3-K) and phospholipase-C**γ** (PLC**γ**), which trigger protein kinase C (PKC) and a calcium flux. The downstream effect of this activating signalling cascade varies between immune cells (Fig**.** 1 **c**).

In contrast to activating FcγRs, FcγRIIB contains an intracellular immunoreceptor tyrosine-based inhibitory motif (ITIM). Cross-linking of FcγRIIB with activating FcγR leads to ITIM phosphorylation by Src kinases, recruiting inositol phosphatases, most notably SHIP1, to neutralise activating signals [36]. Thus, activation and inhibitory FcγRs are co-expressed on the majority of immune cells, and their relatively level of expression allows the cell to modulate the activation threshold of a cell encountering immune complexes. FcγRIIB dysfunction, therefore, has the potential to mediate numerous inflammatory processes in ABMR, including the persistence of DSA-producing plasma cells in the periphery and the local activation of infiltrating immune cells within the allografts.

## FcγR Function in Immune Cells

A number of immune cells have been implicated in the pathogenesis of ABMR, including neutrophils, macrophages, and NK cells. FcγR cross-linking by IgG IC are known to profoundly impact the function of these cells. Furthermore, human endothelial cells can also express FcγRs [37].

### Neutrophils

Human neutrophils constitutively express FcγRIIA and FcγRIIIB, a GPI-linked receptor. Non-activated neutrophils express FcγRIIB2 mRNA [23, 38] but minimal cell-surface levels of FcγRIIB2 [39•]. Similarly, in mouse neutrophils, there is low *fcgr2b* mRNA in bone marrow and blood neutrophils, but expression is significantly increased following activation [40]. Cross-linking of activating FcγRs on neutrophils leads to phagocytosis, cytokine and superoxide production, increased neutrophil adhesion to endothelial cells and neutrophil extracellular trap formation (NETosis) [41–46].

### Macrophages

Macrophages are myeloid cells specialised for phagocytosis that may be tissue-resident (including Kupffer cells in the liver and alveolar macrophages in the lungs) or may differentiate from newly recruited monocytes during local inflammation. Most tissue-resident macrophages express activating FcγRs (FcγRIIA and FcγRIIIA) and FcγRIIB, with the balance tipped in favour of activating FcγR expression. Engagement of activating FcγRs in macrophages results in phagocytosis and cytokine release (including tumour necrosis factor (TNF), IL-6, IL-1α and neutrophil chemoattractants) [47], and the magnitude of this response is controlled by FcγRIIB [48–51].

### Dendritic Cells

DCs express FcγRIIA and FcγRIIIA but in contrast to macrophages, in immature DCs, expression of the inhibitory FcγRIIB dominates. DC maturation signals, such as LPS or IFN-γ, down-regulate FcγRIIB such that IgG-opsonised antigen may be rapidly internalised by activating FcγRs and processed for presentation to T cells, and results in the production of inflammatory cytokines [26, 52, 53]. Furthermore, IgG immune complexes promote DC migration along lymphatics [54••]. FcγRIIB expression on DCs suppresses IC-mediated pro-inflammatory cytokine release, T cell stimulation and migration [55, 54••, 56].

### NK Cells

FcγRIIC and FcγRIIIA expression by NK cells is required for antibody-dependent cellular cytotoxicity (ADCC), whereby cytotoxic granules are released to kill IgG-opsonised cells, but these cells do not express the inhibitory FcγRIIB [57, 58]. As well as ADCC, NK cells undergo IFN-γ release following FcγR cross-linking.

### B Cells and Plasma Cells

FcγRIIB is the only FcγR expressed by B cells, where it cross-links to the B cell receptor (BCR) to increase the B cell activation threshold and suppress antibody production [20]. Furthermore, direct cross-linking of FcγRIIB on the surface of mature B cells and bone marrow-resident plasma cells can directly mediate apoptosis, thereby limiting the peripheral pool of antibody-producing cells [59].

### Endothelial Cells

DSAs can directly mediate endothelial cell activation and proliferation via binding to surface MHC [60, 61]. These effects may be further augmented by simultaneous binding to FcγRs, increasing the expression of adhesion molecules that allow leukocyte recruitment [62]. FcγRI and FcγRII expression on cultured human aortic endothelial cells was shown to mediate IgG internalisation, cytokine production, upregulation of adhesion molecules and activation by CRP in vitro [63]. Furthermore, FcγRIIB has been implicated in the pathogenesis of obesity-induced hypertension, via IgG-mediated attenuation of endothelial NO synthase activity [64]. The extent of FcγR expression on renal endothelial cells is less clear [65]. However, TNF-α and IFN-γ enhance FcγR expression by human endothelial cells in vitro, and this may have added importance in the context of allograft rejection [37].

## FcγRs and ABMR

There are a number of lines of evidence to suggest that FcγRs may mediate inflammation in ABMR as follows:

1. *Mouse models*—Mice deficient in activating FcγRs are protected from antibody-mediated autoimmune pathology, whilst those deficient in the inhibitory receptor FcγRIIB have more aggressive disease [1, 20]. FcγRIIB-deficient mice have been subjected to a murine cardiac allograft model (BM12 organs into C57BL/6 mice). In this model, a chronic vasculopathy is observed, analogous to that in human hearts with chronic rejection, which is driven by autoantibody production. FcγRIIB-deficient mice demonstrated elevated autoantibody production and more severe arteriopathy [66•]. These data are consistent with the known role for FcγRIIB in regulating B cells, but this study did not dissect the relative effect of FcγRIIB on B cells versus myeloid cells. Of note, myeloid-specific FcγRIIB deficiency is sufficient to exacerbate tissue inflammation in a model of antibody-mediated glomerulonephritis [67].
2. *Histological appearances in ABMR*—Although this represents circumstantial evidence, the classical histological features of acute renal ABMR demonstrate the presence of cells known to express FcγRs, including neutrophils within peritubular capillaries and monocytic infiltration of the endothelium and glomeruli. Indeed, the presence of glomerular monocytes in ABMR was associated with worse outcomes, independent of C4d staining [68, 69] and in cardiac allografts with ABMR, a significantly increased number of macrophages has been observed [70]. NK cells are present in the microvascular endothelium in patients with ABMR, a major site of DSA deposition, where ADCC of endothelial cells may directly contribute to graft rejection. Furthermore, NK cell-derived IFNγ (a cytokine known to be produced by NK cells upon FcγR cross-linking) has been implicated in driving a positive feedback loop, in which HLA expression on endothelial cells is enhanced, resulting in further DSA deposition and local immune cell activation [71, 72].

Expanded lymphatic vasculature and mononuclear cell aggregation, including tertiary lymphoid organs have been observed within rejected allografts [73]. IgG immune complexes can induce VEGF-A production by macrophages, driving lymphangiogenesis in vivo, and represents another potential mechanism by which DSA might impact allograft rejection [74].

3. *Transcriptomic signatures in ABMR*—*FCGR3A* transcripts are enriched within renal transplant biopsies, and correlate with the presence of DSA and ABMR. Given the enrichment of other NK cell-associated transcripts, this supports the role of ADCC within chronically rejecting allografts [71, 72, 75•]. FcγRIIIA is also expressed by myeloid cells, and an increase in some macrophage-associated transcripts has also been observed in ABMR, including *CX3CR1* and *IL1B*, suggesting a potential contribution to FcγR-mediated inflammation within allografts.
4. *Genetic association studies in transplantatio* ***n***—A number of SNPs have been identified in both activating and inhibitory FcγRs (Table 1). This region of the genome is also subject to copy number variation. A non-synonymous SNP in FcγRIIA (rs1801274) encodes a histidine to arginine amino acid substitution in the extracellular domain of the receptor (FcγRIIA-H131R). This is associated with a significant reduction in the IgG binding affinity of the receptor. In particular, FcγRIIA-131H is the only human FcγR that binds IgG2 effectively, whilst FcγRIIA-131R binds IgG2 weakly. A SNP in FcγRIIIA *(*rs396991), encoding a valine for a phenylalanine at amino acid 158 in the extracellular domain of the receptor (FcγRIIIA-F158V) also significantly impacts IgG binding. FcγRIIIA-158V has higher affinity for IgG1 and IgG3 than FcγRIIIA-158F [15, 27].

**Table 1:** Polymorphisms in human FcγRs

Receptor	Alleles	Effect
FcγRIIA	H/R131	Increased IgG1 and IgG2 affinity (H131)
	FcγRIIA-exon 6	Enhanced cellular activation
FcγRIIB	I/T232	Impaired inhibitory signalling (T232)
	−386G/C −120T/A	Altered *FCGR2B* promoter activity
FcγRIIC	STOP/Q13	Altered cell surface expression of FcγRIIB/C
FcγRIIIA	V/F-158	Reduced antigen affinity (F158)
FcγRIIIB	NA1/NA2/SH	Increased antigen affinity (NA1) Increased surface FcγRIIIB expression

Several groups have examined activating FcγR SNPs in kidney transplant recipients, although the number included in these case-control studies are small [76–80]. Allograft survival was increased in patients with the FcγRIIA-131R/R genotype [76], but in two subsequent studies this genotype was associated with acute rejection [78, 79], the latter postulated to be due to reduced disposal of deposited IgG. No significant association with FcγRIIIA genotype was observed [79]. Similarly, in a larger study of 200 kidney transplant recipients who had lost their grafts, the FcγRIIA-131R/R genotype was associated with early graft loss (<60 months) and shorter graft survival, particularly in patients who were DSA positive [80]. These genetic data are in contrast to cellular studies demonstrating that monocytes from individuals with the FcγRIIA-131H/H genotype adhered more readily to HLA antibody-activated endothelium compared with FcγRIIA-131R/R monocytes [62], an effect most obvious in the presence of IgG2 DSA. The authors propose that the contrasting results relate to the impact of *FCGR* SNPs on the efficacy of induction therapy, but in the study by Valenzuela et al. non-depleting anti-CD25 antibodies were used, which would not be influenced by FcγR polymorphisms [62]. These conflicting results certainly emphasise the need for more accurate phenotyping of patients included in genetic studies of the *FCGR* locus. Ideally this would include not only routine screening for DSA, but also an assessment of the IgG subclass and the glycosylation of IgG, factors that have a profound impact on the functional significance of genetic polymorphisms.

In humans, a number of non-synonymous SNPs have been identified in the *FCGR2B* gene, of which, only one occurs at a notable frequency (rs1050501). This SNP encodes an isoleucine-to-threonine substitution at position 232 within the transmembrane domain of the receptor, resulting in loss of function [81••, 82]. FcγRIIB-232T is a major risk factor for SLE [83]. Indeed, immune cells isolated from FcγRIIB-232T/T homozygous individuals display heightened immune responses to IgG-IC [81••, 84]. However, in a large study of more than 2800 renal transplant recipients, no association was observed between the autoimmune-associated SNP FcγRIIB-232T and allograft or patient survival [85]. While this supports the hypothesis that FcγRIIIA on NK cells may be the prominent driver of chronic ABMR (FcγRIIB is not expressed by NK cells (Fig. 1c), a lack of patient stratification (including an inability to identify patients with DSA or ABMR), may have masked any effects.

## FcγRs and Recipient Survival

### Infection

In murine models, resistance to infection is intimately linked to FcγR activity [86]. Overall, activating receptor SNPs with increased IgG binding (FcγRIIA-131H, FcγRIIIA-158V) are associated with reduced susceptibility to infection [15, 86], whilst a reduction in FcγRIIB activity increases defence against bacterial [50, 81••, 87], mycobacterial [88], viral [89] and parasitic inf﻿ection﻿ [83, 84]. However, the role of human *FCGR* SNPs on susceptibility to post-transplant infections is yet to be completely elucidated.

FcγRIIA can also bind to acute phase response proteins [21•, 22] that can opsonise pathogens. Unlike IgG, FcγRIIA affinity for CRP is actually reduced in individuals homozygous for FcγRIIA-131H and this may influence outcomes in infection. In a study of post-operative infection in liver transplant patients, individuals that were dually homozygous for the FcγRIIA-131H/H, polymorphism and the polymorphism in *FCGR3A* (F/F158 that reduces IgG binding affinity) were susceptible to blood-borne infections and increased mortality. This susceptibility was attributed to a reduced binding and clearance of CRP-opsonised bacteria, resulting in overwhelming infection [90•].

### Malignancy

Malignancies occur at increased frequency in transplant recipients, particularly skin malignancies and those caused by oncogenic viruses, including post-transplant lymphoproliferative disorder (PTLD). Murine models have demonstrated that IgG opsonised tumour antigens may be effectively processed by DCs to induce anti-tumour responses in an FcγR-dependent manner [91, 92, 93••] and that FcγRs may mediate tumour ADCC [16]. Therefore, it is likely that polymorphisms in human FcγR genes may contribute to differential susceptibility and prognosis in patients with post-transplant malignancy. Indeed, in non-transplant patients with B-cell lymphoma, an increased prevalence of the low-affinity FcγRIIA-131R/R genotype was observed in subjects with Epstein-Barr virus latency and with expression of oncogenic latency proteins [94, 95]. There is also a wealth of evidence demonstrating that activating FcγR polymorphisms can profoundly influence the efficacy of therapeutic monoclonal antibodies used for the treatment of malignancies, including the effect of rituximab in lymphomas [96].

### Atherosclerosis

Evidence suggests that antibodies can be both protective and pathogenic; immunisation with oxLDL reduces atherosclerosis in murine models, likely due to the protective effects of oxLDL-specific antibodies [17, 18]. In addition, intravenous immunoglobulin which contains a mixture of polyclonal IgG from multiple human donors, is also protective in animal models of atherosclerosis [97], and this effect is dependent on the Fc region of IgG [98, 99]. In contrast, other studies highlight the potential pathogenicity of antibodies and B cells [19, 100, 101]. Murine models support a role for activating FcγRs in the development of atherosclerosis [102–105] and suggest that the inhibitory receptor FcγRIIB regulates their pathogenic effects; both apo-E and LDLR-deficient mice develop a more severe disease in the absence of FcγRIIB [106, 107]. These data raise the possibility that functionally significant genetic variants of this receptor in humans might contribute to atheroma susceptibility. A significant association with the rs396991 SNP in FcγRIIIA was demonstrated in one study; patients homozygous for the Fc*γ*RIIIA-V158 allele (encoding a receptor with a high affinity for IgG) had a significantly reduced risk of CAD compared with Fc*γ*RIIIA-F158 homozygotes [108].

## Conclusion

**Fig. 2:**
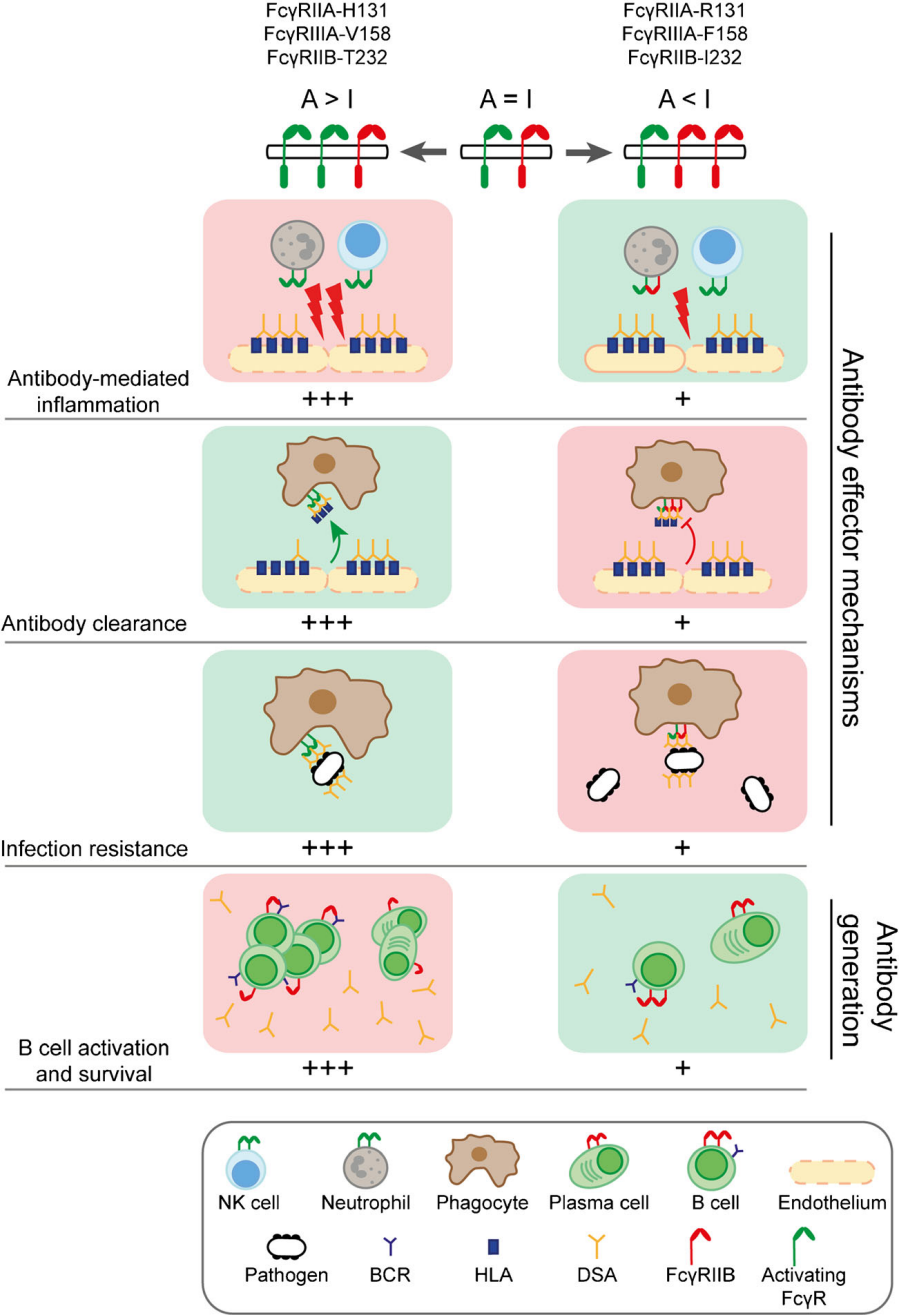
Variation in activating and inhibitory FcγR expression and IgG binding affinity alters inflammation, responses to infection and antibody production**.** SNPs in human *FCGR* genes that lead to higher affinity of activating FcγR for IgG (FcγRIIA-131H, FcγRIIIA-158V) or reduced inhibitory receptor function (FcγRIIB-232T) result in an increased A/I ratio. In the presence of deposited alloantibody, this can drive allograft inflammation through ADCC, cytokine release, and immune cell adhesion, as well as by lowering the threshold for B cell activation and survival in the periphery. However, a high A/I ratio may also promote DSA clearance by mononuclear phagocytes, contributing to the resolution of inflammation and enhance resistance to secondary complications, such as infection and malignancy

FcγRs play an important role in mediating many effector functions of IgG and genetic variation in these receptors and may have a complex impact on outcomes in solid organ transplantation (Fig. 2). The binding of graft-deposited alloantibodies to activating FcγRs on neutrophils, monocytes, macrophages and NK cells may result in inflammation, however could potentially facilitate clearance with minimal inflammation, depending on whether there is co-engagement of FcγRIIB. Furthermore, activating receptor variants with higher affinity for IgG might also improve outcomes in infection and malignancy. This complex balance requires further investigation in solid organ transplantation, particularly before efforts to target these receptors are applied therapeutically.
